# Information on the Harrison Act

**Published:** 1919-03

**Authors:** 


### INFORMATION ON THE HARRISON ACT

For the information of persons authorized to dispense or prescribe narcotics under the Harrison Act, Collector of Internal Revenue Justus S. Wardell, calls attention to Treasury Decision 2200, which reads:

The Act of December 17, 1914, provides that a physician, dentist or veterinary surgeon registered under the provisions of the law, may dispense or prescribe any of the narcotic drugs coming within its scope to patients upon whom he shall ''personally attend'' and ''in the course of professional practice only.''

This office construes the words ''dispensed,'' ''distributer,'' or ''prescribed,'' used in the Act, as synonymous, and that a physician, dentist or veterinary surgeon ''dispenses'' within the meaning of the law when he writes a prescription calling for any narcotic drugs to be filled by a registered dealer.

While the law does not limit or state the quantity of any of the narcotic drugs that may be so dispensed or prescribed at one time, it does provide that it shall be unlawful to obtain by means of order forms any of the aforesaid drugs for any purpose other than the use, sale or distribution thereof in the conduct of a lawful business in said drugs or in the legitimate practice of his profession. Further, that all preparations and remedies containing narcotic drugs coming within the scope of this Act are ''sold, distributed, given away, dispensed or possessed as medicines and not for the purpose of evading the intentions and provisions of this Act,'' and it is further provided that it shall be unlawful for any person not registered to have in his possession or under his control any of the drugs, preparations or remedies, ''which have not been prescribed in good faith by a physician, dentist or veterinary surgeon registered under the Act.''

Therefore, where a physician, dentist or veterinarian prescribes any of the aforesaid drugs in a quantity more than is apparently necessary to meet the immediate needs of a patient in the ordinary case, or where it is for the treatment of an addict or habitue to effect a cure, or for a patient suffering from an incurable or chronic disease, such physician, dentist or veterinary surgeon should indicate on the prescription the purpose for which the unusual quantity of the drug so prescribed is to be used. In cases of treatment of addicts, these prescriptions should show the good faith of the physician in the legitimate practice of his profession by decreasing dosage or reduction of the quantity prescribed from time to time, while, on the other hand, in cases of chronic or incurable diseases such prescription might show an ascending dosage or increased quantity. Registered dealers filling such prescriptions should assure themselves that the drugs are prescribed in good faith for the purpose indicated thereon, and, if there is reason to suspect that the prescriptions are written for the purpose of evading the intentions of the law, such dealers should refuse to fill same.

